# Habitual Intake of Dietary Advanced Glycation End Products Is Not Associated with Arterial Stiffness of the Aorta and Carotid Artery in Adults: The Maastricht Study

**DOI:** 10.1093/jn/nxab097

**Published:** 2021-05-19

**Authors:** Armand Ma Linkens, Simone Jmp Eussen, Alfons Jhm Houben, Abraham A Kroon, Miranda T Schram, Koen D Reesink, Pieter C Dagnelie, Ronald Ma Henry, Marleen van Greevenbroek, Anke Wesselius, Coen Da Stehouwer, Casper G Schalkwijk

**Affiliations:** Department of Internal Medicine, Maastricht University Medical Centre, Maastricht, Netherlands; CARIM School for Cardiovascular Diseases, Maastricht University, Maastricht, Netherlands; CARIM School for Cardiovascular Diseases, Maastricht University, Maastricht, Netherlands; Department of Epidemiology, Maastricht University, Maastricht, Netherlands; Department of Internal Medicine, Maastricht University Medical Centre, Maastricht, Netherlands; CARIM School for Cardiovascular Diseases, Maastricht University, Maastricht, Netherlands; Department of Internal Medicine, Maastricht University Medical Centre, Maastricht, Netherlands; CARIM School for Cardiovascular Diseases, Maastricht University, Maastricht, Netherlands; Department of Internal Medicine, Maastricht University Medical Centre, Maastricht, Netherlands; CARIM School for Cardiovascular Diseases, Maastricht University, Maastricht, Netherlands; Heart and Vascular Center, Maastricht University Medical Centre, Maastricht, Netherlands; CARIM School for Cardiovascular Diseases, Maastricht University, Maastricht, Netherlands; Heart and Vascular Center, Maastricht University Medical Centre, Maastricht, Netherlands; Department of Epidemiology, Maastricht University, Maastricht, Netherlands; Department of Internal Medicine, Maastricht University Medical Centre, Maastricht, Netherlands; CARIM School for Cardiovascular Diseases, Maastricht University, Maastricht, Netherlands; Heart and Vascular Center, Maastricht University Medical Centre, Maastricht, Netherlands; Department of Internal Medicine, Maastricht University Medical Centre, Maastricht, Netherlands; CARIM School for Cardiovascular Diseases, Maastricht University, Maastricht, Netherlands; Department of Complex Genetics and Epidemiology, NUTRIM School for Nutrition and Translational Research in Metabolism, Maastricht University, Maastricht, Netherlands; Department of Internal Medicine, Maastricht University Medical Centre, Maastricht, Netherlands; CARIM School for Cardiovascular Diseases, Maastricht University, Maastricht, Netherlands; Department of Internal Medicine, Maastricht University Medical Centre, Maastricht, Netherlands; CARIM School for Cardiovascular Diseases, Maastricht University, Maastricht, Netherlands

**Keywords:** dietary advanced glycation end products, arterial stiffness, aortic stiffness, carotid stiffness, ultra-performance liquid chromatography tandem mass spectrometry

## Abstract

**Background:**

Advanced glycation end products (AGEs), a heterogeneous group of bioactive compounds, are thought to contribute to arterial stiffness, which in turn is a causal factor in the pathogenesis of stroke, myocardial infarction, and heart failure. Whether AGEs derived from food also contribute to arterial stiffness is not clear.

**Objectives:**

We investigated whether higher intake of dietary AGEs is associated with arterial stiffness.

**Methods:**

In this cross-sectional observational study in 2255 participants of The Maastricht Study (mean ± SD age: 60 ± 8 y, 51% male, mean ± SD BMI: 26.9 ± 4.4 kg/m^2^, *n* = 1326 normal glucose metabolism, *n* = 341 prediabetes, and *n* = 585 type 2 diabetes mellitus), we estimated intake of the dietary AGEs N^ε^-(carboxymethyl)lysine (CML), N^ε^-(1-carboxyethyl)lysine (CEL), and N^δ^-(5-hydro-5-methyl-4-imidazolon-2-yl)-ornithine (MG-H1) by a validated FFQ coupled to our ultra-performance liquid chromatography tandem mass spectrometry dietary AGE database. Arterial stiffness was determined using carotid-femoral pulse wave velocity (cfPWV), carotid distensibility coefficient (DC), and carotid Young's elastic modulus (YEM). We performed multiple linear regression analyses adjusting for potential confounders (demographic, hemodynamic, cardiovascular, and dietary factors).

**Results:**

In the fully adjusted models we observed no statistically significant associations between intake of the dietary AGEs CML, CEL, and MG-H1 and arterial stiffness expressed as cfPWV, carotid DC, and carotid YEM.

**Conclusions:**

In adults aged 40–75 y, habitual intake of the dietary AGEs CML, CEL, and MG-H1 is not associated with arterial stiffness measured as cfPWV, carotid DC, or carotid YEM.

## Introduction

Advanced glycation end products (AGEs) are a heterogeneous group of bioactive compounds formed through the nonenzymatic reaction between reducing sugars and amino acids within proteins and other macromolecules (1). Plasma AGEs, mainly derived from endogenous formation, are associated with arterial stiffness measured as carotid-femoral (i.e., aortic) pulse wave velocity (cfPWV) and carotid distensibility (2–7). Arterial stiffness, in turn, is a causal factor in the pathogenesis of stroke, myocardial infarction, and heart failure (8–12). Endogenously formed AGEs may lead to arterial stiffness by cross-linking collagen within the arterial wall (13) and activating the receptor for AGEs (RAGE), which subsequently leads to low-grade inflammation (14).

In addition to this endogenous formation, AGEs are abundantly present in sugar- and protein-rich food items exposed to dry heat (15). In 450 individuals with an elevated risk of type 2 diabetes mellitus (T2DM) and cardiovascular disease (CVD), a higher habitual intake of these dietary AGEs was associated with higher concentrations of AGEs in plasma (16). Dietary AGEs may have biological effects, because a diet low in AGEs may decrease biomarkers of endothelial dysfunction, low-grade inflammation, and insulin resistance (17). These effects contribute to arterial stiffening (18, 19). Despite this, dietary AGEs were not associated with cfPWV in a cross-sectional study and a randomized controlled trial (RCT) (20, 21). However, in these studies dietary AGEs were estimated using a dietary AGE database based on immunoassay methods and comprised only 1 dietary AGE (22). Furthermore, the sample sizes in both studies were small and only aortic stiffness was assessed.

In view of these considerations, we tested the hypothesis that habitual intake of the specific and well-characterized dietary AGEs N^ε^-(carboxymethyl)lysine (CML), N^ε^-(1-carboxyethyl)lysine (CEL), and N^δ^-(5-hydro-5-methyl-4-imidazolon-2-yl)-ornithine (MG-H1) is associated with arterial stiffness measured both at the aorta and at the carotid artery in a population-based cohort.

## Methods

### Study design and population

**FIGURE 1:**
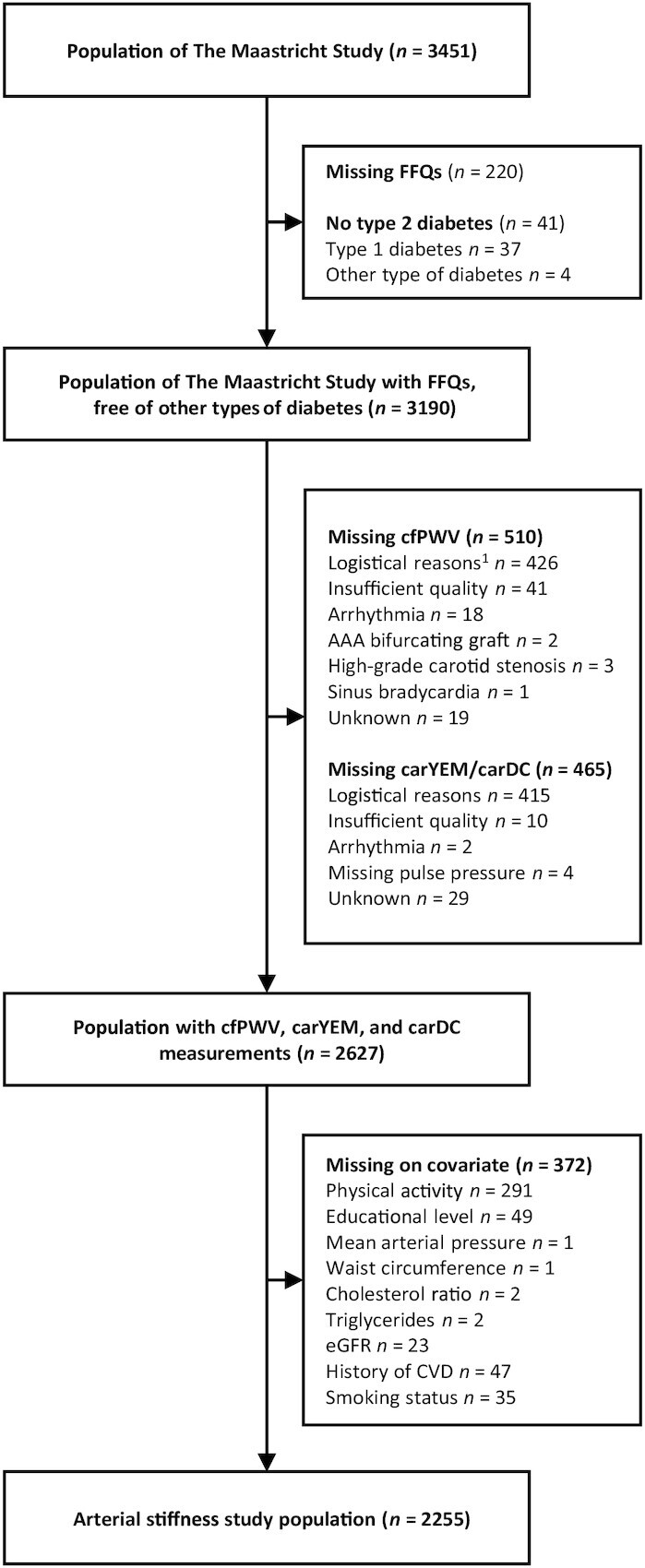
Selection of participants from The Maastricht Study cohort. Missings are not mutually exclusive. ^1^Logistical reasons: no equipment available, no trained researcher available, technical failure. AAA, abdominal aorta aneurysm; carDC, carotid distension coefficient; carYEM, carotid Young's elastic modulus; cfPWV, carotid-femoral pulse wave velocity; CVD, cardiovascular disease; eGFR, estimated glomerular filtration rate.

We used data from The Maastricht Study, an observational prospective population-based cohort study. The rationale and methodology have been described previously (23). In brief, the study focuses on the etiology, pathophysiology, complications, and comorbidities of T2DM and is characterized by an extensive phenotyping approach. Eligible for participation were all individuals aged between 40 and 75 y and living in the southern part of the Netherlands. Participants were recruited through mass-media campaigns and from the municipal registries and the regional Diabetes Patient Registry via mailings. Recruitment was stratified according to known T2DM status for reasons of efficiency. The study has been approved by the institutional medical ethical committee (NL31329.068.10) and the Netherlands Health Council under the Dutch “Law for Population Studies” (Permit 131088-105234-PG). All participants gave written informed consent. The examinations of each participant were performed within a time window of 3 mo. The present report includes cross-sectional data from the first 3451 participants who completed the baseline survey between November 2010 and September 2013. **Figure 1** shows the selection of participants.

### Food intake and dietary AGEs

We assessed dietary intake by a validated 253-item FFQ (24). This FFQ contains 101 questions on consumption with a reference period of 1 y. The FFQ collected information on the intake of major food groups. All participants filled out the FFQ after their first visit to the study center. Those with implausible energy intake (men: <800 or >4200 kcal/d; women: <500 or >3500 kcal/d) were excluded from analyses.

Dietary AGE intake was determined by coupling consumption of food items within the FFQ to our dietary AGE database (25). In this database, 3 major AGEs, CML, CEL, and MG-H1, were quantified in protein fractions using highly specific ultra-performance liquid chromatography tandem mass spectrometry (UPLC-MS/MS). In total, >200 commonly consumed Western food products are included within this database. For each participant, AGE intake was estimated as described previously (16). Some of the food products within the FFQ were not analyzed for AGEs. AGE contents of these specific products were estimated by matching these products to other comparable products. Only upon return of the FFQ were participants informed about their glucose metabolism status. As a result, a possible change in dietary habits due to this information will not be reflected within the results from the FFQ.

### Measurements of central arterial stiffness

All measurements were carried out by trained vascular technicians unaware of the participants’ clinical or diabetes mellitus status. Measurements took place in a quiet, temperature-controlled room (21–23°C) and were performed in the supine position, after 10 min of rest. Participants were asked to refrain from smoking and drinking coffee, tea, or alcoholic beverages for 3 h before measurements. Participants were allowed to have a light meal (breakfast and lunch). Talking or sleeping was not allowed during the examination. A 3-lead electrocardiogram (ECG) was recorded continuously during the measurements to facilitate automatic signal processing. During arterial stiffness measurement, repeated brachial systolic, diastolic, and mean arterial pressures were obtained at 5-min intervals, using an oscillometric device (Accutorr Plus, Datascope, Inc.). The time-averages of the systolic, diastolic, and mean arterial pressures were used in the analysis.

### cfPWV

We measured cfPWV according to recent guidelines (26) with the use of applanation tonometry (SphygmoCor, Atcor Medical). Pressure waveforms were determined at the right common carotid and right common femoral arteries. The difference in the time of pulse arrival from the R-wave of the ECG between the 2 sites (transit time) was determined with the intersecting tangents algorithm. The pulse wave travel distance was calculated as 80% of the direct straight distance (measured with an infantometer) between the 2 arterial sites. cfPWV was defined as travelled distance/transit time. We used the median of 3 consecutive cfPWV recordings in the analyses.

We assessed the reproducibility of cfPWV measurements in 12 individuals (6 men, mean ± SD age: 60.8 ± 6.8 y, 6 individuals with T2DM) who were examined by 2 observers on 2 occasions spaced 1 wk apart. The intra- and interobserver intraclass correlation coefficients were 0.87 and 0.69, respectively.

### Indexes of carotid stiffness

For indexes of carotid stiffness, we measured at the left common carotid artery (10 mm proximal to the carotid bulb), with the use of an ultrasound scanner equipped with a 7.5-MHz linear probe (MyLab 70, Esaote Europe BV). This set-up enables the measurement of diameter, distension, and intima–media thickness (IMT) as described previously (27, 28). Briefly, during the ultrasound measurements, a B-mode image based on 19 M-lines was displayed on-screen. An online echo-tracking algorithm showed real-time anterior and posterior wall displacements. The multiple M-line recordings were composed of 19 simultaneous recordings at a frame rate of 498 Hz. The distance between the M-line recording positions was 0.96 mm; thus, a total segment of 18.24 mm of each artery was covered by the scan plane. For offline processing, the radiofrequency signal was acquired by a dedicated computer-based system (ART.LAB, Esaote Europe BV) with a sampling frequency of 50 MHz. Data processing was performed in MatLab version 7.5 (Mathworks). Distension waveforms were obtained from the radiofrequency data by wall tracking, as described in Hermeling et al. (27). We defined carotid IMT as the distance of the posterior wall from the leading edge interface between lumen and intima to the leading edge interface between media and adventitia (28). We used the median diameter, median distension, and median IMT of 3 recordings in the analyses.

Data analysis was done by quantifying the local arterial elastic properties through the calculation of the following indexes (29):

1. carotid distensibility coefficient (DC) = (2Δ*D* × *D* + Δ*D*^2^)/(PP × *D*^2^) (10^−3^/kPa);
2. carotid Young's elastic modulus (YEM) = *D*/(IMT × DC) (10^3^ kPa)

where *D* is the arterial diameter; Δ*D* is the distension; and PP the pulse pressure. Local carotid PP was estimated according to the calibration method described by Kelly and Fitchett (30), with the use of carotid tonometry waveforms as adapted by Van Bortel et al. (31). This method assumes a constant difference between mean arterial pressure (MAP) and diastolic pressure along the arterial tree. PP can then be calculated at a carotid artery (PPcar) from the uncalibrated carotid pressure waveform using the formula: PPcar = PPcar_uncalibrated_ × (Kbrach/Kcar_uncalibrated_), in which K is defined as (MAP − diastolic pressure). For the carotid artery, diastolic pressure and MAP are calculated as the minimum and the area under the tonometry waveform divided by time, respectively. The carotid DC reflects the inverse of arterial stiffness at operating pressure. The carotid YEM reflects the stiffness of the arterial wall material at operating pressure.

Note that increased values of cfPWV or of carotid YEM, or decreased values of carotid DC, indicate increased central arterial stiffness.

### Glucose metabolism status

To determine glucose metabolism, all participants (except those who used insulin and/or had fasting glucose >11.0 mmol/L) underwent a standardized 7-point oral-glucose-tolerance test (OGTT) after an overnight fast. Blood samples were taken at baseline and at 15, 30, 45, 60, 90, and 120 min after ingestion of a 75-g glucose drink. For safety reasons, participants with a fasting glucose concentration >11.0 mmol/L, as determined by a finger prick, did not undergo the OGTT. For these individuals, fasting glucose concentration and information about diabetes mellitus medication use were used to determine glucose metabolism status. Glucose metabolism status was categorized according to the WHO 2006 criteria into normal glucose tolerance, impaired fasting glucose, impaired glucose tolerance, and T2DM (32). For this study, we defined having impaired fasting glucose and/or impaired glucose tolerance as prediabetes.

### Covariates

Smoking status, history of CVD, and physical activity were assessed by a questionnaire. Smoking status was categorized into never, former, and current smoker. Waist circumference, total cholesterol, HDL cholesterol, LDL cholesterol, triglycerides, fasting plasma glucose, and glycosylated hemoglobin were determined as described elsewhere (23). Estimated glomerular filtration rate (eGFR) was computed with the Chronic Kidney Disease Epidemiology Collaboration formula, using serum creatinine and cystatin C (33). Information on the use of lipid-modifying and/or antihypertensive medication—that is, generic names, doses, and frequencies—was collected during an in-person medication interview. Details on the collection of 24-h ambulatory blood pressure and accelerometer data are provided in the **Supplemental Methods**.

### Statistical methods

Analyses were conducted using SPSS version 25 for Windows (IBM Corporation). To provide an overview on how participant characteristics differ by dietary AGE intake, baseline characteristics are shown for the total study population and stratified by a dietary AGE score that represents an individual's overall AGE intake. Because we measured 3 AGEs in food items which differ in abundance, we first calculated *z* scores for all individual dietary AGEs, which were then averaged into a single dietary AGE score. Participants that were excluded from the analyses owing to missing covariates were compared with the included participants by means of an ANOVA or chi-square test, as appropriate.

We performed multiple linear regression to investigate the association between dietary AGEs (expressed standardized on a continuous scale) and indexes of arterial stiffness (unstandardized). Three regression models were fitted. In model 1, we adjusted for age (y), sex (male or female), glucose metabolism status (normal glucose metabolism, prediabetes, or T2DM—owing to oversampling of individuals with T2DM in The Maastricht Study), and mean heart rate (beats/min) and mean arterial pressure (mm Hg) obtained during vascular measurements because these are important determinants of arterial stiffness. In model 2, we in addition adjusted for CVD risk factors because these may be associated with arterial stiffness: waist circumference (cm), total:HDL cholesterol ratio, triglycerides (mmol/L), use of lipid-lowering medication (%-yes), use of antihypertensive medication (%-yes), prior CVD (%-yes), kidney function (eGFR), smoking status (former, current, never), and alcohol intake (g/d). Finally, in model 3 we in addition adjusted for lifestyle factors that may be associated with dietary intake: total energy intake (kcal/d), the Dutch Healthy Diet index (DHD-index), educational level (low, middle, or high), and physical activity (h/wk). The DHD-index is a measure of diet quality because it assesses adherence to the Dutch dietary guidelines (34). A higher index has been associated with more nutrient-dense diets and lower risk of mortality (35, 36). Crude (model 1) and fully adjusted models (model 3) are presented in the main article, intermediate models (model 2) in the Supplemental Tables.

We also performed multiple linear regression analysis with dietary AGEs expressed categorically. For these analyses, dietary AGEs were divided into quartiles, except for stratified analyses, where dietary AGEs were divided into tertiles for reasons of statistical power. To test for linear trends across categories, an ordinal variable with the median value of dietary AGE intake for each quartile/tertile was entered in the regression models. For tables regarding these analyses, see the Supplementary data.

In addition, we performed interaction analyses for age, sex, glucose metabolism status, and kidney function by adding interaction terms in our model.

We performed several sensitivity analyses. To test the robustness of our results we explored associations between dietary AGEs and arterial stiffness while excluding participants with history of a cardiovascular event, T2DM, or use of antihypertensive medication, because these participants might have altered their diet based on dietary advice from a health care professional. Next, we explored possible confounding of antihypertensive medication after further specification into renin-angiotensin-aldesterone-system inhibitors and other types of antihypertensive medication. We also used ambulatory 24-h blood pressure measurements (heart rate and mean arterial pressure) instead of blood pressure measurements obtained during the vascular measurements. In addition, we replaced physical activity data attained from the CHAMPS questionnaire (37) for accelerometer data (ActivPAL). Finally, to assess whether the observed associations were dependent on the food source of AGEs, we performed separate analyses of AGEs from the largest contributing food sources.

β-Coefficients are shown with their 95% CIs. *P* values < 0.05 were considered statistically significant.

## Results

### Population characteristics

**Table 1** shows characteristics of the population with available data on dietary AGEs, arterial stiffness, and covariates. At a mean ± SD intake of 2187 ± 600 kcal, mean ± SD daily intakes of the dietary AGEs CML, CEL, and MG-H1 were 3.3 ± 1.1 mg, 3.0 ± 1.2 mg, and 24.4 ± 8.9 mg, respectively. Energy intake was higher and age was slightly lower in the higher dietary AGE quartiles, and these participants consumed more energy as fat and less as protein. In addition, the proportion of men, physical activity, waist circumference, systolic and diastolic blood pressure, and carotid DC were higher in the higher dietary AGE quartiles. The main food groups contributing to AGE intake were cereals, meat, confectionaries, dairy products, and nuts (**Supplemental Figure 1**). Individuals with missing data on vascular measurements or covariates were more likely to have T2DM and to smoke, and individuals with missing data on covariates had slightly higher cfPWV, than individuals included in the study population (**Supplemental Table 1**).

**TABLE 1:** Baseline characteristics of 2255 adults of The Maastricht Study^1^

	Total population (*n* = 2255)	Dietary AGE quartiles (*z* score of all dietary AGEs)^2^			
Characteristics		Q1 (*n* = 564)	Q2 (*n* = 563)	Q3 (*n* = 565)	Q4 (*n* = 563)
Age, y	59.9 ± 8.0	60.7 ± 7.7	60.1 ± 8.3	59.7 ± 7.9	59.2 ± 8.0
Sex, male	1145 (51)	197 (35)	267 (47)	302 (53)	379 (67)
Educational level					
Low	712 (32)	201 (36)	156 (27)	196 (35)	159 (28)
Medium	657 (29)	155 (28)	171 (30)	163 (29)	168 (30)
High	886 (39)	208 (37)	236 (42)	206 (37)	236 (42)
Glucose metabolism status					
Normal glucose metabolism	1327 (59)	317 (56)	337 (60)	339 (60)	334 (59)
Prediabetes	342 (15)	86 (15)	76 (14)	88 (16)	92 (16)
Type 2 diabetes mellitus	586 (26)	161 (29)	150 (27)	138 (24)	137 (24)
Smoking					
Never	788 (35)	185 (33)	208 (37)	195 (35)	200 (36)
Former	1197 (53)	301 (53)	288 (51)	302 (53)	306 (54)
Current	270 (12)	78 (14)	67 (12)	68 (12)	57 (10)
Physical activity, h/wk	14.3 ± 8.1	14.2 ± 8.1	14.5 ± 8.0	13.4 ± 7.2	15.0 ± 8.8
Waist circumference, cm	95.4 ± 13.4	94.3 ± 14.2	94.5 ± 13.1	96.1 ± 12.9	96.7 ± 13.3
24-h systolic blood pressure, mm Hg	118.8 ± 11.9	117.5 ± 11.7	118.2 ± 12.1	119.6 ± 11.6	120.0 ± 11.7
24-h diastolic blood pressure, mm Hg	72.5 ± 7.4	72.5 ± 7.4	72.6 ± 7.1	74.3 ± 7.3	74.5 ± 7.0
Antihypertensive medication, yes	855 (38)	232 (41)	213 (38)	212 (38)	198 (35)
Total-to-HDL cholesterol ratio	3.7 ± 1.3	3.6 ± 1.2	3.6 ± 1.2	3.8 ± 1.1	3.8 ± 1.3
Triglycerides, mmol/L	1.2 [0.9–1.7]	1.2 [0.9–1.7]	1.2 [0.9–1.7]	1.2 [0.9–1.7]	1.2 [0.9–1.7]
Lipid-modifying medication, yes	796 (35)	204 (36)	206 (37)	192 (34)	194 (35)
eGFR, mL · min^−1^ · 1.73 m^−2^	88.2 ± 14.4	87.5 ± 14.3	88.5 ± 14.3	87.8 ± 14.3	89.0 ± 14.8
History of cardiovascular disease, yes	359 (16)	88 (16)	92 (16)	93 (17)	86 (15)
Energy intake, kcal/d	2187 ± 600	1623 ± 372	1993 ± 337	2304 ± 397	2826 ± 508
Carbohydrate, % of energy	42.8 ± 6.2	43.0 ± 7.0	43.1 ± 6.4	42.7 ± 5.8	42.8 ± 5.5
Fat, % of energy	34.3 ± 6.0	32.6 ± 6.8	33.8 ± 5.8	35.1 ± 5.6	35.8 ± 5.1
Protein, % of energy	15.9 ± 2.5	16.5 ± 2.9	16.1 ± 2.6	15.8 ± 2.2	15.3 ± 2.2
Fiber, % of energy	2.5 ± 0.6	2.7 ± 0.7	2.5 ± 0.5	2.5 ± 0.5	2.4 ± 0.5
Alcohol, g/d	8.5 [1.5–18.8]	7.2 [0.6–17.5]	8.4 [1.5–17.6]	8.7 [2.0–17.4]	9.4 [2.5–21.0]
Dutch Healthy Diet Index	83.6 ± 14.6	86.3 ± 14.7	84.8 ± 13.9	82.6 ± 14.7	80.7 ± 14.4
Dietary CML, mg/d	3.3 ± 1.1	2.1 ± 0.5	2.9 ± 0.4	3.5 ± 0.4	4.7 ± 1.0
Dietary CEL, mg/d	3.0 ± 1.2	1.8 ± 0.4	2.5 ± 0.3	3.1 ± 0.4	4.5 ± 1.4
Dietary MG-H1, mg/d	24.4 ± 8.9	15.6 ± 3.1	21.0 ± 2.4	25.7 ± 3.0	35.3 ± 9.3
Carotid-femoral pulse wave velocity, m/s	9.0 ± 2.1	9.1 ± 2.2	9.0 ± 2.2	8.8 ± 1.9	8.9 ± 2.1
Carotid distensibility coefficient, mm^2^/kPa	14.3 ± 5.1	13.8 ± 5.0	14.5 ± 5.2	14.5 ± 5.2	15.6 ± 5.0
Carotid Young's elastic modulus, 10^3^/kPa	0.7 ± 0.3	0.8 ± 0.3	0.7 ± 0.4	0.7 ± 0.4	0.7 ± 0.4

1 Values are means ± SDs, medians [IQRs], or *n* (%). AGE, advanced glycation end product; CEL, N^ε^-(1-carboxyethyl)lysine; CML, N^ε^-(carboxymethyl)lysine; eGFR, estimated glomerular filtration rate; MG-H1, N^δ^-(5-hydro-5-methyl-4-imidazolon-2-yl)-ornithine; Q, quartile.

2 Data are shown without stratification, and according to quartiles of a *z* score representing overall dietary AGE intake.

### Intake of dietary AGEs and arterial stiffness

In continuous analyses, the dietary AGEs CML, CEL, and MG-H1 were not associated with cfPWV, carotid DC, or carotid YEM in any of the models (**Table 2**).

**TABLE 2:** Associations between dietary AGEs and arterial stiffness in 2255 adults of The Maastricht Study^1^

Dietary AGE, SD/d	cfPWV, m/s	Carotid DC, mm^2^/kPa	Carotid YEM, 10^3^/kPa
CML			
Semiadjusted β (95% CI)^2^	−0.05 (−0.12, 0.02)	−0.14 (−0.31, 0.04)	0.01 (−0.01, 0.02)
Fully adjusted β (95% CI)^3^	0.04 (−0.07, 0.15)	−0.16 (−0.43, 0.11)	0.01 (−0.01, 0.03)
CEL			
Semiadjusted β (95% CI)^2^	−0.01 (−0.08, 0.06)	−0.06 (−0.23, 0.11)	0.00 (−0.01, 0.01)
Fully adjusted β (95% CI)^3^	0.05 (−0.04, 0.14)	−0.02 (−0.23, 0.20)	−0.01 (−0.02, 0.01)
MG-H1			
Semiadjusted β (95% CI)^2^	−0.06 (−0.13, 0.01)	−0.13 (−0.30, 0.05)	0.01 (−0.01, 0.02)
Fully adjusted β (95% CI)^3^	0.00 (−0.09, 0.10)	−0.11 (−0.35, 0.13)	0.00 (−0.01, 0.02)

1 AGE, advanced glycation end product; CEL, N^ε^-(1-carboxyethyl)lysine; cfPWV, carotid-femoral pulse wave velocity; CML, N^ε^-(carboxymethyl)lysine; DC, distension coefficient; MG-H1, N^δ^-(5-hydro-5-methyl-4-imidazolon-2-yl)-ornithine; YEM, Young's elastic modulus.

2 Regression coefficients (β) and 95% CIs represent the change in arterial stiffness measurement per 1-SD change in dietary AGE intake while adjusted for age, sex, glucose metabolism status, heart rate, and mean arterial pressure obtained during vascular measurements.

3 In addition adjusted for waist circumference, total:HDL cholesterol ratio, triglycerides, smoking habits, use of lipid-lowering medication, use of antihypertensive medication, prior CVD, alcohol intake, kidney function, energy intake, educational level, physical activity, and the Dutch Healthy Diet index.

When expressing dietary AGEs as quartiles, overall associations were similar, except for the associations between MG-H1 and cfPWV, and between CEL and carotid YEM (**Supplemental Table 2**). In models adjusted for participant characteristics and cardiovascular factors, MG-H1 intake >28.6 mg/d (Q4) was associated with a lower cfPWV than MG-H1 intake <18.5 mg/d (Q1) (β: −0.20 m/s; 95% CI: −0.40, 0.00 m/s; *P* for linear trend <0.04), but this association lost statistical significance after adjustment for lifestyle factors in the final model (β: −0.04 m/s; 95% CI: −0.32, 0.23 m/s; *P* for linear trend *=* 0.77). Contrarily, carotid YEM was slightly lower among those in Q3 and Q4 of CEL intake (2.8–3.5 mg/d and >3.5 mg/d) than among those in Q1 (<2.2 mg/d) in fully adjusted models (for Q3: β: −0.05 10^3^/kPa; 95% CI: −0.09, −0.01 10^3^/kPa and for Q4: β: −0.05 10^3^/kPa; 95% CI: −0.10, −0.00 10^3^/kPa; *P* for linear trend = 0.05) (Supplemental Table 2).

Of note, in the fully adjusted models greater total energy intake was independently associated with lower cfPWV, but not with carotid DC or carotid YEM (data not shown).

### Intake of dietary AGEs and arterial stiffness in subgroups of glucose metabolism

Tests for interaction revealed that glucose metabolism status significantly modified the associations between dietary AGEs and cfPWV (all *P* for interactions <0.05), but not with carotid YEM and carotid DC (all *P* for interactions >0.05). However, when also stratified for glucose metabolism status dietary AGEs were not associated with cfPWV in the fully adjusted models (**Supplemental Tables 3, 4**). Sex, age, and kidney function showed no significant interactions for the association between dietary AGEs and measurements of arterial stiffness (all *P* for interactions >0.05, data not shown).

### Additional analyses

When excluding participants with T2DM, use of antihypertensive medication, or a known history of CVD, positive associations between dietary AGEs and cfPWV observed earlier in participants with normal glucose metabolism were attenuated (**Supplemental Table 5**). Likewise, although greater intakes of CML and MG-H1 were associated with lower carotid DC and greater carotid YEM in intermediate models, statistical significance was again lost in the fully adjusted models (Supplemental Table 5). Greater intake of CEL was borderline significantly associated with lower carotid YEM in the fully adjusted model (Supplemental Table 5). Categorical analyses were largely in agreement, except for greater carotid DC in tertile 2 of CML intake than in tertile 1 in the fully adjusted model (Supplemental Table 5).

Next, we replaced physical activity obtained from the CHAMPS questionnaire with accelerometer data, which was available in fewer participants. Although physical activity did not act as a confounder in these analyses, participants in the highest quartile of CEL intake no longer showed lower carotid YEM than those in the lowest quartile (**Supplemental Table 6**). Associations between dietary AGEs and carotid DC were unchanged (data not shown). Participants with T2DM in the highest tertile of MG-H1 intake showed lower cfPWV than those in the lowest tertile, even after adjusting for lifestyle factors (**Supplemental Table 7**). All other sensitivity analyses did not materially change the results (data not shown).

Lastly, we investigated associations between dietary AGEs from food groups and arterial stiffness. In line with overall intake of dietary AGEs, dietary AGEs from these subgroups were not associated with cfPWV or carotid YEM (**Supplemental Tables 8, 9**). In contrast, greater intakes of CML and CEL from cereals were associated with lower carotid DC in the fully adjusted models (β: −0.27 mm^2^/kPa; 95% CI: −0.47, −0.07 mm^2^/kPa for CML, and β: −0.23 mm^2^/kPa; 95% CI: −0.42, −0.04 mm^2^/kPa for CEL) (**Supplemental Table 10**). Dietary AGEs from all other food sources were not associated with carotid DC.

## Discussion

This is to our knowledge the first study on intake of the dietary AGEs CML, CEL, and MG-H1 and arterial stiffness in a population-based setting. Results showed no association of dietary AGEs with arterial stiffness measured as either cfPWV, carotid DC, or carotid YEM, after full adjustment for potential confounders.

Our findings are largely in agreement with 2 previous studies in smaller groups. In a cross-sectional study, Di Pino et al. (20) found a positive association between dietary CML and augmentation index, but not cfPWV, in a group of 85 CVD- and complication-free T2DM patients in adjusted analyses. In line with this, in an RCT, no difference in cfPWV was observed in 62 participants with prediabetes who followed a 24-wk dietary regimen either low or standard in dietary AGEs (21). However, some methodological drawbacks may explain these null findings. Especially for the observational study, the small sample size did not enable adequate correction for possible confounders, although this information was available. Also, dietary AGEs in both studies were measured by ELISA (22), a method that has been shown to quantify low-AGE food products as being high in AGEs, and vice versa, when compared to the gold-standard UPLC-MS/MS technique (38). For the intervention trial, it is possible that the difference in dietary AGEs between groups therefore was too small, especially because 1 of the groups was advised to continue their normal dietary habits. Furthermore, only dietary CML was addressed in both studies. We circumvented these methodological limitations by investigating in relation to arterial stiffness not only dietary CML, but also CEL and MG-H1, which were measured in food items by UPLC-MS/MS (25). These dietary AGEs differ in abundance and structure and may therefore also have different biological effects (39). In addition, the sample size in the current study was much larger and included substantial numbers of individuals with normal glucose metabolism, prediabetes, and T2DM.

Although cfPWV is considered the gold-standard method to determine arterial stiffness, it is important to address both aortic and carotid stiffness, because they are affected differently by CVD risk factors (40), but both predict CVD (8, 11). We observed a small but statistically significant inverse association between quartiles of CEL intake and carotid YEM in the fully adjusted models. However, this observation was not consistent in our sensitivity analyses. There were no associations between the other dietary AGEs and carotid YEM or carotid DC.

We previously showed, in a smaller selection of the current population, that protein-bound pentosidine in plasma and skin autofluorescence (SAF) are associated with higher cfPWV (4). Both protein-bound pentosidine in plasma and SAF are regarded as reflections of collagen cross-linking in the vascular wall, which leads to arterial stiffness by directly altering the structural properties of a vessel. In humans, it is unknown whether dietary AGEs accumulate in tissues such as the vascular wall. In mice, dietary AGEs accumulate in several organs, as shown after chronic exposure to labeled dietary AGEs (41). However, the accumulation of dietary AGEs in plasma and tissues is in its free form and not the protein-bound form, which is in line with the hypothesis that dietary AGEs, consumed as whole proteins, undergo digestion and subsequently enter the circulation as free- or peptide-bound AGEs (16). It is unlikely that the free form of AGEs is involved in collagen cross-linking because this process occurs during endogenous formation of AGEs in collagen. Aside from collagen cross-linking, AGEs may be involved in arterial stiffness by activation of RAGE (42, 43). In line with this, mice fed a CML-enriched diet for 9 mo developed arterial stiffness, an observation that was not seen in RAGE-knockout mice fed the same diet (41). However, whether AGE–RAGE interactions play a role in vascular effects of dietary AGEs in humans is a matter of debate. Only protein-bound AGEs have been reported to have affinity for RAGE, whereas free or peptide-bound AGEs do not (44). This, combined with the findings of the present study and those aforementioned, suggests that dietary AGEs do not share the role of endogenously formed AGEs in arterial stiffness. However, dietary AGEs may have implications beyond arterial stiffness. Consequences of a diet high in AGEs include increased plasma markers of inflammation and endothelial dysfunction. An interesting target for further research is the microcirculation: blood vessels with a diameter <150 μm (45). Because 98% of all endothelial surface resides within the microcirculation, dietary AGE–induced inflammation and endothelial dysfunction may have consequences for microvascular function.

The intake of AGEs in the current cohort is comparable with that in other cohorts where dietary AGEs were assessed using UPLC-MS/MS. In individuals at high risk of CVD and T2DM of the CODAM (Cohort on Diabetes and Atherosclerosis Maastricht) study, we estimated mean daily intake of CML, CEL, and MG-H1 at 3.1 ± 1.0 mg/d, 2.3 ± 0.8 mg/d, and 21.7 ± 6.7 mg/d, respectively (16). The contributions of different food groups to overall AGE intake were largely comparable with those measured in a large subcohort of the EPIC (European Prospective Investigation into Cancer and Nutrition) study (46). In addition, analyses stratified for food groups were largely in agreement with analyses using overall dietary AGE intake, suggesting that our observations were not driven by any of the individual food groups. Although we found that greater intake of CML and CEL from cereals was associated with less carotid DC, indicating greater carotid stiffness, this finding should be interpreted with caution. Cereals represent a wide group of grain products that may differ in cardiovascular properties (47). Owing to the absence of an overall pattern in these additional analyses and the large quantity of statistical tests, this may reflect a false positive finding.

Interestingly, adjusting for total energy intake greatly attenuated the associations between dietary AGEs and cfPWV, and greater total energy intake was associated with lower cfPWV in the fully adjusted models, independently of physical activity. This is a surprising result, because it has been shown that lower total energy intake is associated with lower arterial stiffness (48), whereas the opposite has not yet been described. Although we cannot explain the underlying mechanism, this underlines the importance of adjusting for total energy intake in nutritional epidemiology and researchers investigating the role of dietary AGEs in arterial stiffening should consider this association when performing their analyses.

Our study has several strengths. The extensive phenotyping and population-based approach of The Maastricht Study enable adjustment for important confounders. Furthermore, gold-standard methods were applied where possible: AGEs in food items were measured with the highly specific UPLC-MS/MS technique, and arterial stiffness using recent guidelines (49). The Maastricht Study FFQ, although not a gold-standard method, has been validated against 24-h recalls and several biomarkers (24).

Our study also has several limitations. Primarily, the current study is observational and although we adjusted for many potential confounders, we cannot exclude the possibility of residual confounding. In addition, The Maastricht Study mainly includes middle-aged Caucasians. Applying our results to other populations, especially with different dietary habits, should be performed with caution. In addition, roughly one-third of our participants were excluded from the analysis owing to missing information on FFQs, arterial stiffness, or covariates. The main reason for this was missing arterial stiffness measurements due to logistical reasons (mainly that no trained personnel or no devices were available) that occurred by chance. It is therefore unlikely that this has resulted in selection bias to an extent that it has influenced the current results. A drawback of our FFQ and dietary AGE database is that they do not include detailed information about food preparation. Different cooking techniques and heating durations determine to a large extent the quantity of AGEs in food (50). Despite this, the reliability of dietary AGE assessment was increased by the addition of several high-AGE products in the FFQ (i.e., blood sausage, toasted bread, beef stew). Another limitation is that FFQs are prone to recall bias (51). However, to date there is no reliable biomarker for dietary AGE intake. Furthermore, we previously found an association between dietary AGEs measured with FFQs and free AGEs in plasma in a different cohort, indicating that the combination of an FFQ and our dietary AGE database can be used to assess habitual dietary AGE intake and subsequently rank participants according to their AGE intake.

In conclusion, habitual intake of the dietary AGEs CML, CEL, and MG-H1 was not associated with either cfPWV, carotid DC, or carotid YEM in these cross-sectional analyses. Additional RCTs in which dietary AGEs are accurately assessed by UPLC-MS/MS and the shortcomings of an FFQ are circumvented are needed to fully elucidate the role of dietary AGEs in arterial stiffening. Furthermore, the implications of dietary AGEs for other outcomes, such as microvascular function, deserve further investigation.

## Supplementary Material

nxab097_Supplemental_File

## Notes

The Maastricht Study was supported by the European Regional Development Fund as part of OP-ZUID; the province of Limburg; Ministry of Economic Affairs of the Netherlands grant 31O.041; Stichting de Weijerhorst; the Pearl String Initiative Diabetes; the Cardiovascular Center Maastricht; Cardiovascular Research Institute Maastricht; School for Nutrition, Toxicology, and Metabolism (NUTRIM); Stichting Annadal; Health Foundation Limburg; and by unrestricted grants from Janssen, Novo Nordisk, and Sanofi. We thank the Dutch Diabetes Research Foundation and ZonMw for their financial support.

Author disclosures: The authors report no conflicts of interest.

Supplemental Methods, Supplemental Figure 1, and Supplemental Tables 1–10 are available from the “Supplementary data” link in the online posting of the article and from the same link in the online table of contents at https://academic.oup.com/jn/.

Abbreviations used: AGE, advanced glycation end product; CEL, N^ε^-(1-carboxyethyl)lysine; cfPWV, carotid-femoral pulse wave velocity; CML, N^ε^-(carboxymethyl)lysine; CVD, cardiovascular disease; DC, distensibility coefficient; DHD-index, Dutch Healthy Diet index; ECG, electrocardiogram; eGFR, estimated glomerular filtration rate; IMT, intima–media thickness; MAP, mean arterial pressure; MG-H1, N^δ^-(5-hydro-5-methyl-4-imidazolon-2-yl)-ornithine; OGTT, oral-glucose-tolerance test; PP, pulse pressure; RAGE, receptor for advanced glycation end products; RCT, randomized controlled trial; SAF, skin autofluorescence; T2DM, type 2 diabetes mellitus; UPLC-MS/MS, ultra performance liquid chromatography tandem mass spectrometry; YEM, Young's elastic modulus.
