# Supplementary material for: Habitual Intake of Dietary Advanced Glycation End Products Is Not Associated with Arterial Stiffness of the Aorta and Carotid Artery in Adults: The Maastricht Study
Source: J Nutr. 2021 May 19;151(7):1886–93. doi: 10.1093/jn/nxab097 (PMC8245866; doi:10.1093/jn/nxab097)
Supplement: nxab097_Supplemental_File [file nxab097_supplemental_file.doc]

**Supplementary methods**

**24-hour Ambulatory blood pressure measurement**

Ambulatory blood pressure was measured with ambulatory 24-hour BP monitoring (WatchBP O3, Microlife AG, Switzerland). Cuffs were applied to the participants’ non-dominant arm. Measurements were programmed for every 15 minutes during daytime (08.00–23.00 hours) and every 30 minutes during the night (23.00–08.00 hours), for a total of 24 hours. As quality criteria, mean 24-hour blood pressure measurements were only calculated if more than 14 valid measurements at daytime and more than 7 valid measurements at night were available, based on recommendations of the British Hypertension Society (1). 24-hour ambulatory heart rate, and 24-hour ambulatory mean arterial pressure (MAP, defined as aDBP + (0.412 x aPP)) (2) were calculated based on hourly averages (3).

**Accelerometer data**

Physical activity was measured using the activPAL3 physical activity monitor (PAL Technologies, Glasgow, UK). The activPAL3 is a small (53 × 35 × 7 mm), lightweight (15 g) triaxial accelerometer that records movement in the vertical, anteroposterior and mediolateral axes, and also determines posture (sitting or lying, standing and stepping) based on acceleration information. The device was attached directly to the skin on the front of the right thigh with transparent 3M Tegaderm tape, after the device had been waterproofed using a nitrile sleeve. Participants were asked to wear the accelerometer for 8 consecutive days, without removing it at any time. To avoid inaccurately identifying non-wear time, participants were asked not to replace the device once removed. Data were uploaded using the activPAL software and processed using customized software written in MATLAB R2013b (MathWorks, Natick, MA, USA). Data from the first day were excluded from the analysis because participants performed physical function tests at the research centre after the device was attached. In addition, data from the final wear day providing ≤14 waking hours of data were excluded from the analysis. Participants were included if they provided at least 1 valid day (>14 h of waking data).

**Supplementary Figures**

| 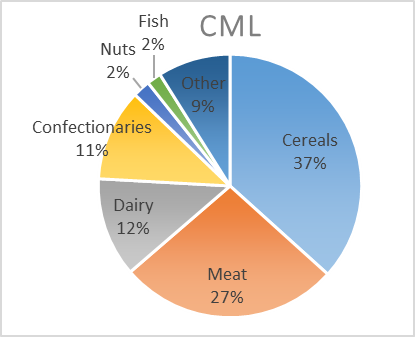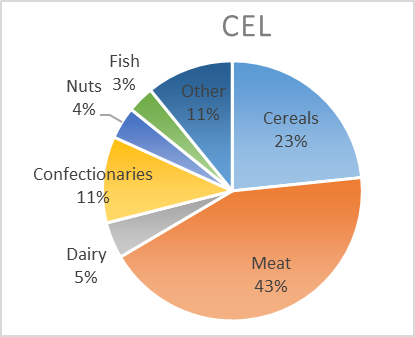  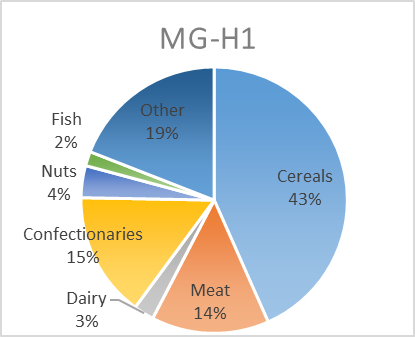 |
| --- |
| **Supplemental Figure 1** Contribution of food groups to AGE intake in 2255 adults of The Maastricht Study. Abbreviations: AGE, advanced glycation endproduct; CEL, N^ε^-(1-carboxyethyl)lysine; CML, N^ε^-(carboxymethyl)lysine; MG-H1, N^δ^-(5-hydro-5-methyl-4-imidazolon-2-yl)-ornithine. |

**Supplementary Tables**

| **Supplemental Table 1** Characteristics of participants included in the analyses compared to those excluded. | | | |
| --- | --- | --- | --- |
| **Characteristics** | **Arterial stiffness population (n=2255)** | **Excluded due to**  **missing (n=1196)** | ***P*-value^1^** |
| Age (years) | 59.9 ± 8.0 | 59.5 ± 8.8 | 0.14 |
| Sex (% male) | 1145 (51%) | 630 (53%) | 0.29 |
| Education level (*n*, %) |  |  | <0.01 |
| Low | 712 (32%) | 423 (38%) |  |
| Medium | 657 (29%) | 297 (26%) |  |
| High | 886 (39%) | 402 (36%) |  |
| Glucose metabolism status (*n ,*%) |  |  | <0.01 |
| Normal glucose metabolism | 1327 (59%) | 597 (50%) |  |
| Prediabetes | 342 (15%) | 169 (14%) |  |
| Type 2 diabetes mellitus | 586 (26%) | 389 (33%) |  |
| Other^2^ | 0 (0%) | 41 (3%) |  |
| Smoking (*n*, %) |  |  | <0.01 |
| Never | 788 (35%) | 384 (34%) |  |
| Former | 1197 (15%) | 562 (49%) |  |
| Current | 270 (26%) | 200 (17%) |  |
| Physical activity (h/week) | 14.3 ± 8.1 | 13.8 ± 8.3 | 0.13 |
| Waist circumference (cm) | 95.4 ± 13.4 | 97.0 ± 14.5 | <0.01 |
| 24-hour Systolic blood pressure (mmHg) | 118.8 ± 11.9 | 119.6 ± 11.6 | 0.10 |
| 24-hour Diastolic blood pressure (mmHg) | 72.5 ± 7.4 | 73.5 ± 6.7 | 0.80 |
| Anti-hypertensives (*n* yes, %) | 855 (38%) | 537 (45%) | <0.01 |
| Total-to-HDL cholesterol ratio | 3.7 ± 1.2 | 3.6 ± 1.2 | 0.27 |
| Triacylglycerides (mmol/L) | 1.2 (0.9-1.7) | 1.2 (0.9-1.8) | 0.46 |
| Lipid-modifying medication (*n* yes*, %*) | 796 (35%) | 463 (39%) | <0.05 |
| eGFR (mL(min·1.73m^2^)) | 88.2 ± 14.4 | 88.0 ± 15.9 | 0.67 |
| History of cardiovascular disease (*n* yes, %) | 359 (16%) | 209 (19%) | 0.06 |
| Energy intake (kcal/day) | 2188 ± 600 | 2167 ± 622 | 0.39 |
| Carbohydrate (% of energy) | 42.8 ± 6.2 | 42.9 ± 6.2 | 0.94 |
| Fat (% of energy) | 34.3 ± 6.0 | 34.4 ± 6.2 | 0.78 |
| Protein (% of energy) | 15.9 ± 2.5 | 16.1 ± 2.8 | 0.07 |
| Fiber (% of energy) | 2.5 ± 0.6 | 2.5 ± 0.6 | 0.99 |
| Alcohol (g/day) | 8.5 (1.5-18.8) | 8.0 (1.0-18.4) | 0.10 |
| Dutch Healthy Diet Index | 83.6 ± 14.6 | 82.4 ± 15.0 | 0.03 |
| Dietary CML (mg/day) | 3.3 ± 1.1 | 3.3 ±1.1 | 0.41 |
| Dietary CEL (mg/day) | 3.0 ± 1.2 | 3.0 ± 1.2 | 0.85 |
| Dietary MG-H1 (mg/day) | 24.4 ± 8.9 | 24.1 ± 8.3 | 0.37 |
| Carotid-femoral Pulse Wave Velocity (m/s) | 9.0 ± 2.1 | 9.2 ± 2.4 | <0.01 |
| Carotid Distensibility Coefficient (mm^2^/kPa) | 14.3 ± 5.1 | 14.3 ± 5.2 | 0.73 |
| Carotid Young’s Elastic Modulus (10^3^/kPa) | 0.7 ± 0.3 | 0.8 ± 0.5 | 0.03 |
| Data are presented as means ± SD, medians (interquartile range), or frequencies (*n,* %), as appropriate. Abbreviations: CEL, N^ε^-(1-carboxyethyl)lysine; CML, N^ε^-(carboxymethyl)lysine; eGFR, estimated glomerular filtration rate; MG-H1, N^δ^-(5-hydro-5-methyl-4-imidazolon-2-yl)-ornithine.  ^1^ Comparisons between groups were performed with ANOVA or X^2^, as appropriate.  ^2^ Other types of diabetes: Type 1 diabetes mellitus, surgery or medicine-induced diabetes mellitus. | | | |

| **Supplemental Table 2** Associations between dietary AGEs and arterial stiffness in 2255 adults of The Maastricht Study. | | | | | | |
| --- | --- | --- | --- | --- | --- | --- |
|  | **Continuous** | **Quartile 1**  (ref) *n*=564 | **Quartile 2**  *n*=564 | **Quartile 3**  *n*=564 | **Quartile 4**  *n*=564 | ***P*_trend_** |
| **cfPWV (m/s)** |  |  |  |  |  |  |
| CML | SD/day | <2.5 mg/day | 2.5-3.1 mg/day | 3.1-3.9 mg/day | >3.9 mg/day |  |
| Model 1 β (95% CI)^1^ | -0.05 (-0.12,0.02) | 0 | -0.21 (-0.40,-0.01) | -0.17 (-0.37,0.03) | -0.19 (-0.38,0.02) | 0.14 |
| Model 2 β (95% CI)^2^ | -0.06 (-0.13,0.02) | 0 | -0.21 (-0.41,-0.01) | -0.18 (-0.37,0.02) | -0.20 (-0.40,0.01) | 0.12 |
| Model 3 β (95% CI)^3^ | 0.04 (-0.07,0.15) | 0 | -0.14 (-0.34,0.07) | -0.05 (-0.28,0.18) | 0.02 (-0.27,0.31) | 0.68 |
| CEL | SD/day | <2.2 mg/day | 2.2-2.8 mg/day | 2.8-3.5 mg/day | >3.5 mg/day |  |
| Model 1 β (95% CI)^1^ | -0.01 (-0.08,0.06) | 0 | -0.02 (-0.22,0.18) | -0.16 (-0.36,0.04) | -0.12 (-0.32,0.09) | 0.17 |
| Model 2 β (95% CI)^2^ | -0.02 (-0.09,0.05) | 0 | -0.01 (-0.21,0.18) | -0.18 (-0.37,0.02) | -0.13 (-0.33,0.07) | 0.12 |
| Model 3 β (95% CI)^3^ | 0.05 (-0.04,0.14) | 0 | 0.05 (-0.16,0.25) | -0.08 (-0.29,0.15) | 0.07 (-0.21,0.30) | 0.91 |
| MG-H1 | SD/day | <18.5 mg/day | 18.5-23.1 mg/day | 23.1-28.6 mg/day | >28.6 mg/day |  |
| Model 1 β (95% CI)^1^ | -0.06 (-0.13,0.01) | 0 | -0.11 (-0.31,0.08) | -0.18 (-0.38,0.02) | -0.21 (-0.42,-0.02) | 0.03 |
| Model 2 β (95% CI)^2^ | -0.06 (-0.13,0.01) | 0 | -0.10 (-0.30,0.10) | -0.18 (-0.38,0.02) | -0.20 (-0.40,-0.00) | 0.04 |
| Model 3 β (95% CI)^3^ | 0.00 (-0.09,0.10) | 0 | -0.05 (-0.26,0.16) | -0.09 (-0.31,0.14) | -0.04 (-0.32,0.23) | 0.77 |
| **carDC (mm^2^/kPa)** |  |  |  |  |  |  |
| CML | SD/day | <2.5 mg/day | 2.5-3.1 mg/day | 3.1-3.9 mg/day | >3.9 mg/day |  |
| Model 1 β (95% CI)^1^ | -0.14 (-0.31,0.04) | 0 | 0.29 (-0.19,0.77) | 0.09 (-0.39,0.57) | -0.22 (-0.71,0.28) | 0.23 |
| Model 2 β (95% CI)^2^ | -0.12 (-0.30,0.05) | 0 | 0.30 (-0.18,0.78) | 0.13 (-0.35,0.61) | -0.16 (-0.65,0.34) | 0.32 |
| Model 3 β (95% CI)^3^ | -0.16 (-0.43,0.11) | 0 | 0.32 (-0.18,0.83) | 0.16 (-0.40,0.71) | -0.11 (-0.81,0.60) | 0.57 |
| CEL | SD/day | <2.2 mg/day | 2.2-2.8 mg/day | 2.8-3.5 mg/day | >3.5 mg/day |  |
| Model 1 β (95% CI)^1^ | -0.06 (-0.23,0.11) | 0 | 0.01 (-0.47,0.49) | 0.14 (-0.34,0.62) | 0.10 (-0.40,0.59) | 0.64 |
| Model 2 β (95% CI)^2^ | -0.05 (-0.23,0.12) | 0 | 0.00 (-0.47,0.48) | 0.19 (-0.29,0.67) | 0.13 (-0.36,0.63) | 0.49 |
| Model 3 β (95% CI)^3^ | -0.02 (-0.23,0.20) | 0 | 0.12 (-0.38,0.61) | 0.38 (-0.15,0.91) | 0.45 (-0.17,1.08) | 0.12 |
| MG-H1 | SD/day | <18.5 mg/day | 18.5-23.1 mg/day | 23.1-28.6 mg/day | >28.6 mg/day |  |
| Model 1 β (95% CI)^1^ | -0.13 (-0.30,0.05) | 0 | 0.27 (-0.21,0.74) | -0.11 (-0.59,0.37) | -0.14 (-0.63,0.35) | 0.31 |
| Model 2 β (95% CI)^2^ | -0.12 (-0.30,0.05) | 0 | 0.24 (-0.24,0.72) | -0.10 (-0.58,0.38) | -0.12 (-0.60,0.37) | 0.38 |
| Model 3 β (95% CI)^3^ | -0.11 (-0.35,0.13) | 0 | 0.30 (-0.20,0.80) | -0.02 (-0.57,0.54) | 0.04 (-0.63,0.71) | 0.86 |
| **carYEM (10^3^/kPa)** |  |  |  |  |  |  |
| CML | SD/day | <2.5 mg/day | 2.5-3.1 mg/day | 3.1-3.9 mg/day | >3.9 mg/day |  |
| Model 1 β (95% CI)^1^ | 0.01 (-0.01,0.02) | 0 | -0.02 (-0.05,0.02) | -0.00 (-0.04,0.03) | 0.02 (-0.02,0.05) | 0.28 |
| Model 2 β (95% CI)^2^ | 0.01 (-0.01,0.02) | 0 | -0.02 (-0.05,0.02) | -0.01 (-0.04,0.03) | 0.01 (-0.02,0.05) | 0.37 |
| Model 3 β (95% CI)^3^ | 0.01 (-0.01,0.03) | 0 | -0.02 (-0.05,0.02) | -0.01 (-0.05,0.04) | 0.01 (-0.04,0.06) | 0.54 |
| CEL | SD/day | <2.2 mg/day | 2.2-2.8 mg/day | 2.8-3.5 mg/day | >3.5 mg/day |  |
| Model 1 β (95% CI)^1^ | 0.00 (-0.01,0.01) | 0 | -0.03 (-0.06,0.01) | -0.03 (-0.07,0.01) | -0.02 (-0.06,0.02) | 0.36 |
| Model 2 β (95% CI)^2^ | -0.00 (-0.01,0.01) | 0 | -0.03 (-0.06,0.01) | -0.03 (-0.07,0.00) | -0.02 (-0.06,0.01) | 0.27 |
| Model 3 β (95% CI)^3^ | -0.01 (-0.02,0.01) | 0 | -0.03 (-0.07,0.00) | -0.05 (-0.09,-0.01) | -0.05 (-0.10,-0.00) | 0.05 |
| MG-H1 | SD/day | <18.5 mg/day | 18.5-23.1 mg/day | 23.1-28.6 mg/day | >28.6 mg/day |  |
| Model 1 β (95% CI)^1^ | 0.01 (-0.01,0.02) | 0 | -0.01 (-0.05,0.02) | -0.01 (-0.03,0.04) | -0.01 (-0.03,0.05) | 0.73 |
| Model 2 β (95% CI)^2^ | 0.01 (-0.01,0.02) | 0 | -0.01 (-0.05,0.03) | 0.00 (-0.03,0.04) | 0.01 (-0.03,0.05) | 0.47 |
| Model 3 β (95% CI)^3^ | 0.00 (-0.01,0.02) | 0 | -0.01 (-0.05,0.03) | 0.00 (-0.04,0.04) | 0.00 (-0.05,0.05) | 0.79 |
| Data are shown according to quartiles of AGE intake and AGEs expressed continuously. Abbreviations: AGE, advanced glycation endproduct; CarDC, carotid distension coefficient; CarYEM, carotid Young’s Elastic Modulus; CEL, N^ε^-(1-carboxyethyl)lysine; cfPWV, carotid-to-femoral pulse wave velocity; CML, N^ε^-(carboxymethyl)lysine; MG-H1, N^δ^-(5-hydro-5-methyl-4-imidazolon-2-yl)-ornithine.  ^1^ Regression coefficients (β) and 95% CI represent the difference in arterial stiffness (in m/s for cfPWV, mm^2^/kPa for carotid DC, and 10^3^/kPa for carotid YEM) per 1-SD change in dietary AGE intake or for a dietary AGE quartile compared to the reference quartile while adjusted for age, sex, glucose metabolism status, and heart rate and mean arterial pressure obtained during vascular measurements.  ^2^ Additionally adjusted for: waist circumference, total/high-density lipoprotein ratio, triglycerides, smoking habits, use of lipid-lowering medication, use of antihypertensive medication, prior CVD, alcohol intake, and kidney function.  ^3^ Additionally adjusted for: energy intake, educational level, physical activity and the Dutch Healthy Diet index. | | | | | | |

| **Supplemental Table 3** Associations between dietary AGEs and cfPWV (m/s) in adults of The Maastricht Study by glucose metabolism status | | | | | |
| --- | --- | --- | --- | --- | --- |
| **Dietary AGE**  (SD/day) | **NGM**  *n*=1327 | **Prediabetes**  *n*=342 | **T2DM**  *n*=586 | ***P*_interaction_^3^** | ***P*_interaction_^4^** |
| CML |  |  |  |  |  |
| Semi-adjusted β (95% CI)^1^ | 0.04 (-0.05,0.13) | -0.21 (-0.42,-0.02) | -0.10 (-0.26,0.05) |  |  |
| Fully-adjusted β (95% CI)^2^ | 0.09 (-0.05,0.23) | -0.04 (-0.32,0.24) | 0.04 (-0.20,0.28) | <0.01 | 0.01 |
| CEL |  |  |  |  |  |
| Semi-adjusted β (95% CI)^1^ | 0.07 (-0.02,0.16) | -0.11 (-0.29,0.07) | -0.06 (-0.21,0.09) |  |  |
| Fully-adjusted β (95% CI)^2^ | 0.10 (-0.02,0.21) | 0.00 (-0.21,0.22) | 0.03 (-0.15,0.21) | <0.01 | 0.02 |
| MG-H1 |  |  |  |  |  |
| Semi-adjusted β (95% CI)^1^ | -0.01 (-0.10,0.08) | -0.11 (-0.28,0.07) | -0.11 (-0.26,0.05) |  |  |
| Fully-adjusted β (95% CI)^2^ | -0.03 (-0.16,0.11) | 0.04 (-0.17,0.26) | 0.01 (-0.20,0.22) | 0.01 | 0.05 |
| Abbreviations: AGE, advanced glycation endproduct; CEL, N^ε^-(1-carboxyethyl)lysine; cfPWV, carotid-to-femoral pulse wave velocity; CML, N^ε^-(carboxymethyl)lysine; MG-H1, N^δ^-(5-hydro-5-methyl-4-imidazolon-2-yl)-ornithine; NGM, normal glucose metabolism; T2DM, type 2 diabetes mellitus.  ^1^ Regression coefficients (β) and 95% CI represent the change in cfPWV (in m/s) per 1-SD change in dietary AGE intake while adjusted for age, sex, and heart rate and mean arterial pressure obtained during vascular measurements  ^2^ Additionally adjusted for waist circumference, total/high-density lipoprotein ratio, triglycerides, smoking habits, use of lipid-lowering medication, use of antihypertensive medication, prior CVD, alcohol intake, kidney function, energy intake, educational level, physical activity and the Dutch Healthy Diet index.  ^3^ Interaction for NGM versus prediabetes, only tested in the fully-adjusted model  ^4^ Interaction for NGM versus T2DM, only tested in the fully-adjusted model | | | | | |

| **Supplemental Table 4** Associations between dietary AGEs as tertiles and cfPWV (m/s) in adults of The Maastricht Study by glucose metabolism status. | | | | | |
| --- | --- | --- | --- | --- | --- |
|  | **Continuous** | **Tertile 1**  (ref) | **Tertile 2** | **Tertile 3** | ***P*_trend_** |
| **CML** |  |  |  |  |  |
| Normal glucose metabolism | SD/day | <2.7 mg/day | 2.7-3.6 mg/day | >3.6 mg/day |  |
| Sample size | 1327 | 442 | 443 | 442 |  |
| Model 1 β (95% CI)^1^ | 0.04 (-0.05,0.13) | 0 | 0.07 (-0.12,0.26) | 0.11 (-0.09,0.31) | 0.28 |
| Model 2 β (95% CI)^2^ | 0.05 (-0.04,0.14) | 0 | 0.08 (-0.11,0.27) | 0.13 (-0.07,0.33) | 0.20 |
| Model 3 β (95% CI)^3^ | 0.09 (-0.05,0.23) | 0 | 0.12 (-0.09,0.33) | 0.20 (-0.07,0.48) | 0.16 |
| Prediabetes | SD/day | <2.8 mg/day | 2.8-3.7 mg/day | >3.7 mg/day |  |
| Sample size | 342 | 114 | 114 | 114 |  |
| Model 1 β (95% CI)^1^ | -0.21 (-0.42,-0.02) | 0 | -0.34 (-0.85,0.16) | -0.50 (-1.01,0.01) | 0.05 |
| Model 2 β (95% CI)^2^ | -0.21 (-0.41,-0.02) | 0 | -0.33 (-0.84,0.19) | -0.49 (-1.01,0.01) | 0.06 |
| Model 3 β (95% CI)^3^ | -0.04 (-0.32,0.24) | 0 | -0.15 (-0.71,0.40) | -0.07 (-0.76,0.62) | 0.86 |
| T2DM | SD/day | <2.7 mg/day | 2.7-3.6 mg/day, | >3.6 mg/day |  |
| Sample size | 586 | 195 | 196 | 195 |  |
| Model 1 β (95% CI)^1^ | -0.10 (-0.26,0.05) | 0 | -0.34 (-0.75,0.06) | -0.39 (-0.80,0.03) | 0.08 |
| Model 2 β (95% CI)^2^ | -0.12 (-0.28,0.03) | 0 | -0.38 (-0.79,0.02) | -0.44 (-0.85,-0.02) | 0.05 |
| Model 3 β (95% CI)^3^ | 0.04 (-0.20,0.28) | 0 | -0.22 (-0.67,0.23) | -0.17 (-0.76,0.42) | 0.63 |
| **CEL** |  |  |  |  |  |
| Normal glucose metabolism | SD/day | <2.4 mg/day | 2.4-3.4 mg/day | >3.4 mg/day |  |
| Sample size | 1327 | 442 | 443 | 442 |  |
| Model 1 β (95% CI)^1^ | 0.07 (-0.02,0.16) | 0 | 0.03 (-0.16,0.22) | 0.02 (-0.18,0.21) | 0.89 |
| Model 2 β (95% CI)^2^ | 0.07 (-0.02,0.16) | 0 | 0.01 (-0.18,0.20) | 0.01 (-0.19,0.20) | 0.97 |
| Model 3 β (95% CI)^3^ | 0.10 (-0.02,0.21) | 0 | 0.00 (-0.20,0.20) | -0.03 (-0.27,0.22) | 0.83 |
| Prediabetes | SD/day | <2.4 mg/day | 2.4-3.3 mg/day | >3.3 mg/day |  |
| Sample size | 342 | 114 | 114 | 114 |  |
| Model 1 β (95% CI)^1^ | -0.11 (-0.29,0.07) | 0 | -0.18 (-0.68,0.32) | -0.47 (-0.98,0.03) | 0.06 |
| Model 2 β (95% CI)^2^ | -0.13 (-0.32,0.06) | 0 | -0.19 (-0.69,0.31) | -0.52 (-1.04,-0.01) | 0.04 |
| Model 3 β (95% CI)^3^ | 0.00 (-0.21,0.22) | 0 | -0.04 (-0.57,0.49) | -0.19 (-0.82,0.45) | 0.55 |
| T2DM | SD/day | <2.3 mg/day | 2.3-3.2 mg/day | >3.2 mg/day |  |
| Sample size | 586 | 195 | 196 | 195 |  |
| Model 1 β (95% CI)^1^ | -0.06 (-0.21,0.09) | 0 | -0.13 (-0.54,0.27) | -0.36 (-0.77,0.05) | 0.08 |
| Model 2 β (95% CI)^2^ | -0.08 (-0.22,0.07) | 0 | -0.11 (-0.51,0.30) | -0.35 (-0.76,0.07) | 0.10 |
| Model 3 β (95% CI)^3^ | 0.03 (-0.15,0.21) | 0 | 0.06 (-0.37,0.50) | -0.05 (-0.58,0.48) | 0.83 |
| **MG-H1** |  |  |  |  |  |
| Normal glucose metabolism | SD/day | <20.5 mg/day | 20.5-26.7 mg/day | >26.7 mg/day |  |
| Sample size | 1327 | 442 | 443 | 442 |  |
| Model 1 β (95% CI)^1^ | -0.01 (-0.10,0.08) | 0 | -0.09 (-0.29,0.13) | -0.01 (-0.20,0.19) | 0.97 |
| Model 2 β (95% CI)^2^ | 0.00 (-0.09,0.09) | 0 | -0.08 (-0.27,0.11) | 0.02 (-0.18,0.22) | 0.76 |
| Model 3 β (95% CI)^3^ | -0.03 (-0.16,0.11) | 0 | -0.09 (-0.30,0.11) | -0.00 (-0.27,0.26) | 0.93 |
| Prediabetes | SD/day | <19.9 mg/day | 19.9-26.7 mg/day | >26.7 mg/day |  |
| Sample size | 342 | 114 | 114 | 114 |  |
| Model 1 β (95% CI)^1^ | -0.11 (-0.28,0.07) | 0 | -0.25 (-0.75,0.25) | -0.46 (-0.96,0.04) | 0.08 |
| Model 2 β (95% CI)^2^ | -0.11 (-0.28,0.07) | 0 | -0.21 (-0.72,0.29) | -0.42 (-0.92,0.09) | 0.11 |
| Model 3 β (95% CI)^3^ | 0.04 (-0.17,0.26) | 0 | -0.00 (-0.55,0.55) | 0.06 (-0.64,0.75) | 0.87 |
| T2DM | SD/day | <19.5 mg/day | 19.5-25.9 mg/day | >25.9 mg/day |  |
| Sample size | 586 | 195 | 196 | 195 |  |
| Model 1 β (95% CI)^1^ | -0.11 (-0.26,0.05) | 0 | 0.09 (-0.31,0.50) | -0.41 (-0.81,-0.00) | 0.03 |
| Model 2 β (95% CI)^2^ | -0.12 (-0.27,0.04) | 0 | 0.06 (-0.35,0.47) | -0.42 (-0.83,-0.02) | 0.03 |
| Model 3 β (95% CI)^3^ | 0.01 (-0.20,0.22) | 0 | 0.18 (-0.26,0.62) | -0.20 (-0.73,0.33) | 0.39 |
| Abbreviations: AGE, advanced glycation endproduct; CEL, N^ε^-(1-carboxyethyl)lysine; cfPWV, carotid-to-femoral pulse wave velocity; CML, N^ε^-(carboxymethyl)lysine; MG-H1, N^δ^-(5-hydro-5-methyl-4-imidazolon-2-yl)-ornithine.  ^1^ Regression coefficients (Β) and 95% CI represent the difference in cfPWV (in m/s) per 1-SD change in dietary AGE intake or for a dietary AGE tertile compared to the reference tertile while adjusted for age, sex and heart rate and mean arterial pressure obtained during vascular measurements.  ^2^ Additionally adjusted for waist circumference, total/high-density lipoprotein ratio, triglycerides, smoking habits, use of lipid-lowering medication, use of antihypertensive medication, prior CVD, alcohol intake, and kidney function.  ^3^ Additionally adjusted for energy intake, educational level, physical activity and the Dutch Healthy Diet index. | | | | | |

| **Supplemental Table 5** Associations between dietary AGEs and arterial stiffness in 1128 apparently healthy adults of The Maastricht Study | | | | | |
| --- | --- | --- | --- | --- | --- |
|  | **Continuous**  *n*=1128 | **Tertile 1**  (Ref) *n*=376 | **Tertile 2**  *n*=376 | **Tertile 3**  *n*=376 | ***P*_trend_** |
| **cfPWV (m/s)** |  |  |  |  |  |
| CML | SD/day | <2.7 mg/day | 2.7-3.6 mg/day | >3.6 mg/day |  |
| Model 1 β (95% CI)^1^ | -0.02 (-0.12,0.07) | 0 | 0.08 (-0.13,0.29) | 0.00 (-0.22,0.22) | 0.95 |
| Model 2 β (95% CI)^2^ | -0.02 (-0.11,0.08) | 0 | 0.08 (-0.13,0.29) | 0.01 (-0.21,0.23) | 0.97 |
| Model 3 β (95% CI)^3^ | 0.04 (-0.11,0.19) | 0 | 0.14 (-0.09,0.36) | 0.14 (-0.16,0.44) | 0.40 |
| CEL | SD/day | <2.4 mg/day | 2.4-3.2 mg/day | >3.2 mg/day |  |
| Model 1 β (95% CI)^1^ | 0.03 (-0.06,0.13) | 0 | 0.03 (-0.18,0.24) | -0.05 (-0.26,0.16) | 0.60 |
| Model 2 β (95% CI)^2^ | 0.03 (-0.07,0.13) | 0 | 0.02 (-0.19,0.22) | -0.07 (-0.28,0.15) | 0.51 |
| Model 3 β (95% CI)^3^ | 0.09 (-0.04,0.22) | 0 | 0.04 (-0.18,0.26) | -0.01 (-0.28,0.26) | 0.90 |
| MG-H1 | SD/day | <20.4 mg/day | 20.4-26.6 mg/day | >26.6 mg/day |  |
| Model 1 β (95% CI)^1^ | -0.04 (-0.14,0.06) | 0 | -0.07 (-0.28,0.14) | -0.06 (-0.27,0.16) | 0.65 |
| Model 2 β (95% CI)^2^ | -0.03 (-0.13,0.07) | 0 | -0.07 (-0.28,0.13) | -0.04 (-0.26,0.17) | 0.74 |
| Model 3 β (95% CI)^3^ | -0.00 (-0.15,0.14) | 0 | -0.03 (-0.26,0.19) | 0.04 (-0.25,0.33) | 0.75 |
| **carDC (mm^2^/kPa)** |  |  |  |  |  |
| CML | SD/day | <2.7 mg/day | 2.7-3.6 mg/day | >3.6 mg/day |  |
| Model 1 β (95% CI)^1^ | -0.31 (-0.58,-0.03) | 0 | 0.43 (-0.17,1.03) | -0.63 (-1.25,-0.01) | 0.03 |
| Model 2 β (95% CI)^2^ | -0.29 (-0.56,-0.02) | 0 | 0.46 (-0.13,1.06) | -0.57 (-1.20,0.05) | 0.04 |
| Model 3 β (95% CI)^3^ | -0.04 (-0.48,0.40) | 0 | 0.67 (0.02,1.32) | -0.11 (-0.98,0.75) | 0.64 |
| CEL | SD/day | <2.4 mg/day | 2.4-3.2 mg/day | >3.2 mg/day |  |
| Model 1 β (95% CI)^1^ | -0.17 (-0.45,0.11) | 0 | -0.01 (-0.60,0.59) | -0.09 (-0.71,0.52) | 0.75 |
| Model 2 β (95% CI)^2^ | -0.12 (-0.41,0.16) | 0 | 0.05 (-0.55,0.64) | 0.02 (-0.59,0.64) | 0.95 |
| Model 3 β (95% CI)^3^ | 0.18 (-0.19,0.54) | 0 | 0.36 (-0.27,0.98) | 0.74 (-0.02,1.50) | 0.06 |
| MG-H1 | SD/day | <20.4 mg/day | 20.4-26.6 mg/day | >26.6 mg/day |  |
| Model 1 β (95% CI)^1^ | -0.34 (-0.62,-0.06) | 0 | -0.01 (-0.60,0.59) | -0.44 (-1.05,0.18) | 0.14 |
| Model 2 β (95% CI)^2^ | -0.33 (-0.61,-0.05) | 0 | -0.00 (-0.59,0.59) | -0.41 (-1.03,0.20) | 0.17 |
| Model 3 β (95% CI)^3^ | -0.08 (-0.49,0.34) | 0 | 0.33 (-0.31,0.97) | 0.30 (-0.52,1.12) | 0.51 |
| **carYEM (10^3^/kPa)** |  |  |  |  |  |
| CML | SD/day | <2.7 mg/day | 2.7-3.6 mg/day | >3.6 mg/day |  |
| Model 1 β (95% CI)^1^ | 0.02 (-0.00,0.03) | 0 | -0.02 (-0.06,0.02) | 0.04 (0.01,0.08) | 0.03 |
| Model 2 β (95% CI)^2^ | 0.02 (-0.00,0.03) | 0 | -0.02 (-0.06,0.02) | 0.04 (-0.00,0.08) | 0.04 |
| Model 3 β (95% CI)^3^ | -0.01 (-0.04,0.02) | 0 | -0.04 (-0.08,0.01) | 0.00 (-0.05,0.06) | 0.80 |
| CEL | SD/day | <2.4 mg/day | 2.4-3.2 mg/day | >3.2 mg/day |  |
| Model 1 β (95% CI)^1^ | 0.01 (-0.01,0.03) | 0 | 0.01 (-0.03,0.05) | 0.01 (-0.03,0.05) | 0.63 |
| Model 2 β (95% CI)^2^ | 0.00 (-0.02,0.02) | 0 | 0.00 (-0.04,0.04) | 0.00 (-0.04,0.04) | 0.96 |
| Model 3 β (95% CI)^3^ | -0.02 (-0.04,0.00) | 0 | -0.02 (-0.06,0.02) | -0.05 (-0.10,0.00) | 0.06 |
| MG-H1 | SD/day | <20.4 mg/day | 20.4-26.6 mg/day | >26.6 mg/day |  |
| Model 1 β (95% CI)^1^ | 0.02 (-0.00,0.03) | 0 | -0.02 (-0.05,0.02) | 0.02 (-0.02,0.06) | 0.20 |
| Model 2 β (95% CI)^2^ | 0.02 (-0.00,0.03) | 0 | -0.01 (-0.05,0.02) | 0.02 (-0.02,0.06) | 0.22 |
| Model 3 β (95% CI)^3^ | -0.01 (-0.03,0.02) | 0 | -0.04 (-0.08,0.02) | -0.03 (-0.08,0.03) | 0.43 |
| Abbreviations: AGE, advanced glycation endproduct; CarDC, carotid distension coefficient; CarYEM, carotid Young’s Elastic Modulus; CEL, N^ε^-(1-carboxyethyl)lysine; cfPWV, carotid-to-femoral pulse wave velocity; CML, N^ε^-(carboxymethyl)lysine; MG-H1, N^δ^-(5-hydro-5-methyl-4-imidazolon-2-yl)-ornithine.  ^1^ Regression coefficients (Beta) and 95% CI represent the difference in arterial stiffness (in m/s, for cfPWV, mm^2^/kPa for carDC, and 10^3^/kPa for carYEM) per 1-SD change in dietary AGE intake or for a dietary AGE tertile compared to the reference tertile while adjusted for age, sex, glucose metabolism status, and heart rate and mean arterial pressure obtained during vascular measurements.  ^2^ Additionally adjusted for waist circumference, total/high-density lipoprotein ratio, triglycerides, smoking habits, use of lipid-lowering medication, use of antihypertensive medication, prior CVD, alcohol intake, and kidney function.  ^3^ Additionally adjusted for energy intake, educational level, physical activity and the Dutch Healthy Diet index. | | | | | |

| **Supplemental Table 6** Associations between dietary AGEs and carotid YEM in 1912 adults of The Maastricht Study while substituting physical activity obtained from questionnaires for accelerometer data. | | | | | | |
| --- | --- | --- | --- | --- | --- | --- |
| **Dietary**  **AGE** | **Continuous**  *n=*1912 | **Quartile 1**  (ref) *n*=478 | **Quartile 2**  *n*=478 | **Quartile 3**  *n*=478 | **Quartile 4**  *n*=478 | ***P*_trend_** |
| CML | SD/day | <2.5 mg/day | 2.5-3.1 mg/day | 3.1-3.9 mg/day | >3.9 mg/day |  |
| Model 1 β (95% CI)^1^ | 0.01 (-0.01,0.02) | 0 | -0.01 (-0.04,0.03) | 0.01 (-0.02,0.05) | 0.02 (-0.02,0.06) | 0.26 |
| Model 2 β (95% CI)^2^ | 0.01 (-0.01,0.02) | 0 | -0.01 (-0.04,0.03) | 0.01 (-0.02,0.05) | 0.02 (-0.02,0.05) | 0.27 |
| Model 3 β (95% CI)^3^ | 0.01 (-0.01,0,03) | 0 | -0.01 (-0.04,0.03) | 0.02 (-0.03,0.06) | 0.02 (-0.04,0.07) | 0.39 |
| CEL | SD/day | <2.2 mg/day | 2.2-2.8 mg/day | 2.8-3.5 mg/day | >3.5 mg/day |  |
| Model 1 β (95% CI)^1^ | 0.00 (-0.01,0.02) | 0 | -0.02 (-0.06,0.01) | -0.02 (-0.06,0.02) | -0.02 (-0.06,0.02) | 0.50 |
| Model 2 β (95% CI)^2^ | 0.00 (-0.01,0.02) | 0 | -0.02 (-0.06,0.02) | -0.02 (-0.05,0.02) | -0.01 (-0.05,0.02) | 0.60 |
| Model 3 β (95% CI)^3^ | -0.00 (-0.02,0.02) | 0 | -0.03 (-0.06,0.01) | -0.03 (-0.07,0.01) | -0.03 (-0.08,0.01) | 0.24 |
| MG-H1 | SD/day | <18.6 mg/day | 18.6-23.0 mg/day | 23.0-28.5 mg/day | >28.5 mg/day |  |
| Model 1 β (95% CI)^1^ | 0.01 (-0.01,0.02) | 0 | -0.01 (-0.05,0.02) | 0.02 (-0.02,0.06) | 0.00 (-0.03,0.04) | 0.56 |
| Model 2 β (95% CI)^2^ | 0.01 (-0.01,0.03) | 0 | -0.01 (-0.05,0.03) | 0.02 (-0.02,0.06) | 0.01 (-0.03,0.05) | 0.42 |
| Model 3 β (95% CI)^3^ | 0.01 (-0.01,0.03) | 0 | -0.01 (-0.05,0.03) | 0.02 (-0.02,0.06) | 0.01 (-0.05,0.06) | 0.64 |
| Abbreviations: AGE, advanced glycation endproduct; Carotid YEM, carotid Young’s Elastic Modulus; CEL, N^ε^-(1-carboxyethyl)lysine; CML, N^ε^-(carboxymethyl)lysine; MG-H1, N^δ^-(5-hydro-5-methyl-4-imidazolon-2-yl)-ornithine.  ^1^ Regression coefficients (β) and 95% CI represent the difference in carYEM (10^3^/kPa) per SD-change in dietary AGE intake or for categorical analyses, difference for a dietary AGE quartile compared to the reference quartile while adjusted for age, sex, glucose metabolism status and heart rate and mean arterial pressure obtained during vascular measurements.  ^2^ Additionally adjusted for waist circumference, total/high-density lipoprotein ratio, triglycerides, smoking habits, use of lipid-lowering medication, use of antihypertensive medication, prior CVD, alcohol intake, and kidney function.  ^3^ Additionally adjusted for energy intake, educational level, physical activity and the Dutch Healthy Diet index. | | | | | | |

| **Supplemental Table 7** Associations between dietary AGEs and cfPWV in adults of The Maastricht Study, while substituting physical activity obtained from questionnaires for accelerometer data, stratified for glucose metabolism status. | | | | | |
| --- | --- | --- | --- | --- | --- |
| **Dietary AGE** | **Continuous** | **Tertile 1**  (ref) | **Tertile 2** | **Tertile 3** | ***P*trend** |
| **CML** |  |  |  |  |  |
| Total group | SD/day | <2.7 mg/day | 2.7-3.6 mg/day | >3.6 mg/day |  |
| Sample size | 1912 | 637 | 637 | 638 |  |
| Model 1 β (95% CI)^1^ | -0.08 (-0.16, 0.01) | 0 | -0.06 (-0.25,0.13) | -0.19 (-0.39,0.00) | 0.05 |
| Model 2 β (95% CI)^2^ | -0.08 (-0.16,0.00) | 0 | -0.06 (-0.25,0.13) | -0.19 (-0.38,0.01) | 0.06 |
| Model 3 β (95% CI)^3^ | 0.01 (-0.11,0.14) | 0 | 0.02 (-0.19,0.23) | -0.01 (-0.28,0.26) | 0.93 |
| Normal glucose metabolism | SD/day | <2.7 mg/day | 2.7-3.6 mg/day | >3.6 mg/day |  |
| Sample size | 1082 | 360 | 361 | 361 |  |
| Model 1 β (95% CI)^1^ | -0.01 (-0.11,0.10) | 0 | 0.18 (-0.06,0.41) | 0.12 (-0.12,0.37) | 0.68 |
| Model 2 β (95% CI)^2^ | 0.00 (-0.10,0.10) | 0 | 0.13 (-0.08,0.34) | 0.07 (-0.15,0.29) | 0.59 |
| Model 3 β (95% CI)^3^ | 0.05 (-0.12,0.21) | 0 | 0.16 (-0.07,0.39) | 0.15 (-0.16,0.45) | 0.39 |
| Prediabetes | SD/day | <2.8 mg/day | 2.8-3.7 mg/day | >3.7 mg/day |  |
| Sample size | 278 | 95 | 97 | 95 |  |
| Model 1 β (95% CI)^1^ | -0.16 (-0.39,0.07) | 0 | -0.11 (-0.68,0.46) | -0.31 (-0.89,0.26) | 0.28 |
| Model 2 β (95% CI)^2^ | -0.17 (-0.40,0.06) | 0 | -0.05 (-0.64,0.54) | -0.31 (-0.89,0.28) | 0.30 |
| Model 3 β (95% CI)^3^ | -0.03 (-0.35,0.29) | 0 | 0.20 (-0.44,0.85) | 0.22 (-0.57,1.01) | 0.60 |
| T2DM | SD/day | <2.7 mg/day | 2.7-3.6 mg/day | >3.6 mg/day |  |
| Sample size | 543 | 181 | 181 | 181 |  |
| Model 1 β (95% CI)^1^ | -0.11 (-0.27,0.06) | 0 | 0.34 (-0.76,0.08) | -0.46 (-0.89,-0.03) | 0.05 |
| Model 2 β (95% CI)^2^ | -0.12 (-0.28,0.05) | 0 | -0.35 (-0.77,0.08) | -0.47 (0.90,-0.04) | 0.04 |
| Model 3 β (95% CI)^3^ | 0.01 (-0.24,0.27) | 0 | -0.25 (-0.71,0.22) | -0.34 (-0.94,0.26) | 0.28 |
| **CEL** |  |  |  |  |  |
| Total group | SD/day | <2.4 mg/day | 2.4-3.2 mg/day | >3.2 mg/day |  |
| Sample size | 1912 | 637 | 637 | 638 |  |
| Model 1 β (95% CI)^1^ | -0.04 (-0.12,0.04) | 0 | -0.02 (-0.21,0.17) | -0.27 (-0.51,-0.08) | 0.00 |
| Model 2 β (95% CI)^2^ | -0.05 (-0.13,0.04) | 0 | -0.03 (-0.22,0.16) | -0.28 (-0.47,-0.08) | 0.00 |
| Model 3 β (95% CI)^3^ | 0.03 (-0.07,0.13) | 0 | 0.02 (-0.18,0.22) | -0.18 (-0.42,0.06) | 0.11 |
| Normal glucose metabolism | SD/day | <2.4 mg/day | 2.4-3.4 mg/day | >3.2 mg/day |  |
| Sample size | 1082 | 361 | 361 | 360 |  |
| Model 1 β (95% CI)^1^ | 0.04 (-0.06,0.15) | 0 | 0.09 (-0.13,0.29) | -0.00 (-0.22,0.21) | 0.92 |
| Model 2 β (95% CI)^2^ | 0.04 (-0.07,0.14) | 0 | 0.07 (-0.14,0.28) | -0.02 (-0.24,0.20) | 0.80 |
| Model 3 β (95% CI)^3^ | 0.09 (-0.05,0.23) | 0 | 0.07 (-0.16,0.29) | -0.03 (-0.30,0.25) | 0.81 |
| Prediabetes | SD/day | <2.4 mg/day | 2.4-3.3 mg/day | >3.3 mg/day |  |
| Sample size | 278 | 96 | 95 | 96 |  |
| Model 1 β (95% CI)^1^ | -0.09 (-0.29,0.12) | 0 | -0.06 (-0.63,0.50) | -0.49 (-1.05,0.08) | 0.08 |
| Model 2 β (95% CI)^2^ | -0.09 (-0.30,0.12) | 0 | -0.08 (-0.66,0.50) | -0.50 (-1.08,0.08) | 0.08 |
| Model 3 β (95% CI)^3^ | 0.01 (-0.23,0.25) | 0 | 0.06 (-0.55,0.68) | -0.21 (-0.92,0.49) | 0.50 |
| T2DM | SD/day | <2.3 mg/day | 2.3-3.2 mg/day | >3.2 mg/day |  |
| Sample size | 543 | 181 | 181 | 181 |  |
| Model 1 β (95% CI)^1^ | -0.07 (-0.22,0.09) | 0 | -0.21 (-0.63,0.21) | -0.56 (-0.98,-0.13) | 0.01 |
| Model 2 β (95% CI)^2^ | -0.08 (-0.24,0.08) | 0 | -0.17 (-0.59,0.25) | -0.54 (-0.97,-0.11) | 0.01 |
| Model 3 β (95% CI)^3^ | 0.00 (-0.18,0.19) | 0 | -0.10 (-0.55,0.35) | -0.44 (-0.98,0.10) | 0.10 |
| **MG-H1** |  |  |  |  |  |
| Total group | SD/day | <20.1 mg/day | 20.1-26.3 mg/day | >26.3 mg/day |  |
| Sample size | 1912 | 637 | 637 | 638 |  |
| Model 1 β (95% CI)^1^ | -0.10 (-0.18,-0.01) | 0 | -0.12 (-0.31,0.07) | -0.32 (-0.54,-0.11) | <0.01 |
| Model 2 β (95% CI)^2^ | -0.09 (-0.17,-0.01) | 0 | -0.11 (-0.31,0.08) | -0.29 (-0.51,-0.13) | <0.01 |
| Model 3 β (95% CI)^3^ | -0.02 (-0.13,0.09) | 0 | -0.08 (-0.28,0.12) | -0.20 (-0.46,0.06) | 0.12 |
| Normal glucose metabolism | SD/day | <20.5 mg/day | 20.5-26.7 mg/day | >26.7 mg/day |  |
| Sample size | 1082 | 360 | 361 | 361 |  |
| Model 1 β (95% CI)^1^ | -0.06 (-0.16,0.04) | 0 | -0.08 (-0.28,0.14) | -0.05 (-0.26,0.17) | 0.68 |
| Model 2 β (95% CI)^2^ | -0.05 (-0.15,0.06) | 0 | -0.07 (-0.28,0.14) | -0.02 (-0.24,0.19) | 0.86 |
| Model 3 β (95% CI)^3^ | -0.08 (-0.23,0.08) | 0 | -0.08 (-0.31,0.15) | -0.04 (-0.33,0.25) | 0.86 |
| Prediabetes | SD/day | <19.9 mg/day | 19.9-26.7 mg/day | >26.7 mg/day |  |
| Sample size | 278 | 95 | 96 | 96 |  |
| Model 1 β (95% CI)^1^ | -0.06 (-0.26,0.13) | 0 | -0.09 (-0.66,0.48) | -0.37 (-0.94,0.20) | 0.19 |
| Model 2 β (95% CI)^2^ | -0.06 (-0.26,0.14) | 0 | -0.05 (-0.63,0.54) | -0.36 (-0.94,0.22) | 0.21 |
| Model 3 β (95% CI)^3^ | 0.06 (-0.18,0.30) | 0 | 0.16 (-0.47,0.79) | 0.10 (-0.69,0.88) | 0.84 |
| T2DM | SD/day | <19.5 mg/day | 19.5-25.9 mg/day | >25.9 mg/day |  |
| Sample size | 543 | 181 | 181 | 181 |  |
| Model 1 β (95% CI)^1^ | -0.12 (-0.29,0.05) | 0 | -0.11 (-0.53,0.31) | -0.63 (-1.05,-0.21) | <0.01 |
| Model 2 β (95% CI)^2^ | -0.13 (-0.30,0.04) | 0 | -0.09 (-0.52,0.33) | -0.62 (-1.04,-0.20) | <0.01 |
| Model 3 β (95% CI)^3^ | -0.04 (-0.26,0.19) | 0 | -0.08 (-0.53,0.38) | -0.60 (-1.15,-0.05) | 0.02 |
| Abbreviations: AGE, advanced glycation endproduct; CEL, N^ε^-(1-carboxyethyl)lysine; cfPWV, carotid-to-femoral pulse wave velocity; CML, N^ε^-(carboxymethyl)lysine; MG-H1, N^δ^-(5-hydro-5-methyl-4-imidazolon-2-yl)-ornithine.  ^1^ Regression coefficients (Beta) and 95% CI represent the difference in cfPWV (in m/s) per 1-SD change in dietary AGE intake and per change in dietary AGE tertile compared to the reference tertile while adjusted for age, sex, and heart rate and mean arterial pressure obtained during vascular measurements.  ^2^ Additionally adjusted for waist circumference, total/high-density lipoprotein ratio, triglycerides, smoking habits, use of lipid-lowering medication, use of antihypertensive medication, prior CVD, alcohol intake, and kidney function.  ^3^ Additionally adjusted for energy intake, educational level, physical activity, and the Dutch Healthy Diet index. | | | | | |

| **Supplemental Table 8** Associations between dietary AGEs from food groups and cfPWV (m/s) in 2255 adults of The Maastricht Study. | | | |
| --- | --- | --- | --- |
| **AGEs from food groups**  β (95% CI) per 1 SD/day^1^ | **CML** | **CEL** | **MG-H1** |
| Cereals | -0.03 (-0.11,0.05) | -0.01 (-0.07,0.09) | -0.01 (-0.07,0.09) |
| Meat | 0.06 (-0.02,0.14) | 0.06 (-0.02,0.13) | 0.03 (-0.05,0.10) |
| Fish | -0.05 (-0.12,0.03) | -0.04 (-0.12,0.03) | -0.04 (-0.12,0.03) |
| Dairy | 0.04 (-0.03,0.11) | 0.03 (-0.05,0.10) | 0.02 (-0.05,0.10) |
| Confectionaries | -0.05 (-0.12,0.03) | -0.06 (-0.14,0.01) | -0.07 (-0.14,0.01) |
| Nuts | 0.05 (-0.02,0.13) | 0.05 (-0.02,0.13) | 0.05 (-0.02,0.13) |
| Abbreviations: AGE, advanced glycation endproduct; CEL, N^ε^-(1-carboxyethyl)lysine; cfPWV, carotid-to-femoral pulse wave velocity; CML, N^ε^-(carboxymethyl)lysine; MG-H1, N^δ^-(5-hydro-5-methyl-4-imidazolon-2-yl)-ornithine.  ^1^ Regression coefficients (β) and 95% CI represent the difference in cfPWV (m/s) per SD-change in dietary AGE intake while adjusted for age, sex, and glucose metabolism status, heart rate and mean arterial pressure obtained during vascular measurements, waist circumference, total/high-density lipoprotein ratio, triglycerides, smoking habits, use of lipid-lowering medication, use of antihypertensive medication, prior CVD, alcohol intake, and kidney function, energy intake, educational level, physical activity, and the Dutch Healthy Diet index.. | | | |

| **Supplemental Table 9** Associations between dietary AGEs from food groups and carotid YEM (10^3^/kPa) in 2255 adults of The Maastricht Study. | | | |
| --- | --- | --- | --- |
| **AGEs from food groups**  β (95% CI) per 1 SD/day^1^ | **CML** | **CEL** | **MG-H1** |
| Cereals | 0.01 (-0.00,0.03) | 0.01 (-0.01,0.02) | 0.01 (-0.01,0.02) |
| Meat | -0.01 (-0.02,0.01) | -0.01 (-0.02,0.01) | 0.00 (-0.01,0.02) |
| Fish | -0.00 (-0.02,0.01) | -0.01 (-0.02,0.01) | -0.00 (-0.02,0.01) |
| Dairy | 0.00 (-0.01,0.02) | 0.00 (-0.01,0.02) | 0.00 (-0.01,0.01) |
| Confectionaries | -0.00 (-0.02,0.01) | -0.00 (-0.01,0.01) | 0.00 (-0.01,0.01) |
| Nuts | -0.01 (-0.02,0.00) | -0.01 (-0.02,0.00) | -0.01 (-0.02,0.00) |
| Abbreviations: AGE, advanced glycation endproduct; Carotid YEM, carotid Young’s Elastic Modulus; CEL, N^ε^-(1-carboxyethyl)lysine; cfPWV, carotid-to-femoral pulse wave velocity; CML, N^ε^-(carboxymethyl)lysine; MG-H1, N^δ^-(5-hydro-5-methyl-4-imidazolon-2-yl)-ornithine.  ^1^ Regression coefficients (β) and 95% CI represent the difference in carotid YEM (10^3^/kPa) per SD-change in dietary AGE intake while adjusted for age, sex, and glucose metabolism status, heart rate and mean arterial pressure obtained during vascular measurements, waist circumference, total/high-density lipoprotein ratio, triglycerides, smoking habits, use of lipid-lowering medication, use of antihypertensive medication, prior CVD, alcohol intake, and kidney function, energy intake, educational level, physical activity, and the Dutch Healthy Diet index.. | | | |

| **Supplemental Table 10** Associations between dietary AGEs from food groups and carotid DC (mm^2^/kPa) in 2255 adults of The Maastricht Study. | | | |
| --- | --- | --- | --- |
| **AGEs from food groups**  β (95% CI) per 1 SD/day^1^ | **CML** | **CEL** | **MG-H1** |
| Cereals | -0.27 (-0.47,-0.07) | -0.23 (-0.42,-0.04) | -0.17 (-0.37,0.03) |
| Meat | -0.02 (-0.22,0.18) | -0.01 (-0.19,0.17) | -0.11 (-0.28,0.07) |
| Fish | 0.01 (-0.17,0.19) | 0.03 (-0.15,0.21) | 0.00 (-0.18,0.18) |
| Dairy | -0.06 (-0.24,0.12) | -0.06 (-0.25,0.13) | -0.10 (-0.29,0.08) |
| Confectionaries | 0.18 (-0.01,0.36) | 0.14 (-0.04,0.32) | 0.12 (-0.06,0.30) |
| Nuts | 0.06 (-0.13,0.24) | 0.06 (-0.13,0.24) | 0.06 (-0.13,0.24) |
| Abbreviations: AGE, advanced glycation endproduct; Carotid DC, carotid distension coefficient; CEL, N^ε^-(1-carboxyethyl)lysine; cfPWV, carotid-to-femoral pulse wave velocity; CML, N^ε^-(carboxymethyl)lysine; MG-H1, N^δ^-(5-hydro-5-methyl-4-imidazolon-2-yl)-ornithine.  ^1^ Regression coefficients (β) and 95% CI represent the difference in carotid DC (mm^2^/kPa) per SD-change in dietary AGE intake while adjusted for age, sex, and glucose metabolism status, heart rate and mean arterial pressure obtained during vascular measurements, waist circumference, total/high-density lipoprotein ratio, triglycerides, smoking habits, use of lipid-lowering medication, use of antihypertensive medication, prior CVD, alcohol intake, and kidney function, energy intake, educational level, physical activity, and the Dutch Healthy Diet index. | | | |

**Supplemental references**

1. O'Brien E, Coats A, Owens P, Petrie J, Padfield PL, Littler WA, de Swiet M, Mee F. Use and interpretation of ambulatory blood pressure monitoring: recommendations of the British hypertension society. Bmj. 2000 Apr 22;320(7242):1128-34.

2. Meaney E, Alva F, Moguel R, Meaney A, Alva J, Webel R. Formula and nomogram for the sphygmomanometric calculation of the mean arterial pressure. Heart. 2000 Jul;84(1):64.

3. Mancia G, Fagard R, Narkiewicz K, Redon J, Zanchetti A, Bohm M, Christiaens T, Cifkova R, De Backer G, Dominiczak A, et al. 2013 ESH/ESC guidelines for the management of arterial hypertension: the Task Force for the Management of Arterial Hypertension of the European Society of Hypertension (ESH) and of the European Society of Cardiology (ESC). European heart journal. 2013 Jul;34(28):2159-219.
